# Q Fever Severe Pericarditis With Cardiac Tamponade: A Case Report

**DOI:** 10.7759/cureus.34980

**Published:** 2023-02-14

**Authors:** Antonios Pikoulas, Sofia Arapi, Georgia Kosta, Christos Lampropoulos, Ioanna Papaioannou

**Affiliations:** 1 Department of Clinical Therapeutics, National and Kapodistrian University of Athens, School of Medicine, Athens, GRC; 2 Department of Medicine, University of Crete and Foundation for Research and Technology, Heraklion, GRC; 3 Cardiology, G. Gennimatas General Hospital, Athens, GRC; 4 Radiology, Alexandra General Hospital, Athens, GRC; 5 Emergency Department, Alexandra General Hospital, Athens, GRC

**Keywords:** endocarditis, serological diagnosis, pericarditis, q fever, coxiella burnetii

## Abstract

Q fever can present in acute or chronic form with a wide range of clinical symptoms and presentations. Here we report severe pericarditis with cardiac tamponade due to a chronic Coxiella burnetii (C. burnetii) infection. Our report emphasizes and justifies the importance of serological testing for chronic Q fever in patients with unexplained pericarditis, particularly in areas where C. burnetii is endemic.

## Introduction

Q fever is a zoonosis with a worldwide distribution caused by the bacterium Coxiella burnetii (C. burnetii). The term “Q fever” (derived from “query fever”) was suggested in 1937 after an outbreak of febrile illness affecting workers of an abattoir in Queensland, Australia. The causative microorganism C. burnetii is a small, obligate intracellular Gram-negative bacterium. The reservoir of C. burnetii is broad and includes ruminants (goats, sheep, cattle), pets, humans (incidental hosts in most cases), birds, and even arthropods (mainly ticks). Bacteria are excreted in the infected animals’ urine, feces, milk, and birth products. Infection can be acquired by inhalation of contaminated fomites of aerosol from dried animals’ products, direct contact with infected products, and skin bites from arthropod vectors. At the same time, there have also been reports of human-to-human transmission [1]. C. burnetii is highly infectious, and a small number of microorganisms are capable of causing disease [2].

The incubation period ranges from seven to 40 days, depending on the inoculum, with the average period being 15 to 21 days. Most infected individuals remain asymptomatic (approximately 60%), and most symptomatic individuals will only develop mild symptoms without hospitalization [3]. Q fever can present in either acute or chronic form. The most common clinical presentations of acute Q fever are self-limited febrile illness with headaches, atypical pneumonia, and hepatitis (abnormal hepatic enzyme levels with/without hepatomegaly and rarely jaundice). Chronic Q fever refers to spontaneous evolution with a duration of more than six months and a high titer of IgG antibodies against phase I C. burnetii. It usually affects immunocompromised patients or patients with cardiovascular abnormalities (e.g., heart valve defects, congenital heart disease, prosthetic heart valves). The most common clinical presentations are endocarditis, osteoarticular, vascular, and pulmonary chronic infections.

Here we report the case of a woman presenting with fever, cough, and a large pericardial effusion diagnosed with chronic Q fever.

## Case presentation

A 58-year-old woman presented to the emergency room due to low-grade fever, malaise, muscle aches, and non-productive cough for the past five days that evolved into a productive cough with dark-colored sputum, high-grade fever (up to 39^o^C) and diffuse thoracic ache one day before her presentation. The patient had a personal history of hypertension (under irbesartan/hydrochlorothiazide 300/25mg qd and amlodipine 5mg qd) and diabetes mellitus (under alogliptin/metformin 12.5/1000mg bid). Chest auscultation revealed reduced air entry and the presence of crackles at the left lower lobe, while the rest of the physical examination was normal. Vital signs were: heart rate 115bpm, respiratory rate 28/min, blood pressure 140/80mmHg, SpO_2_ 96%, and a body temperature 38.5^o^C. Due to the COVID-19 pandemic, a nasal swab was tested for SARS-CoV-2, and the result was negative.

**Figure 1:**
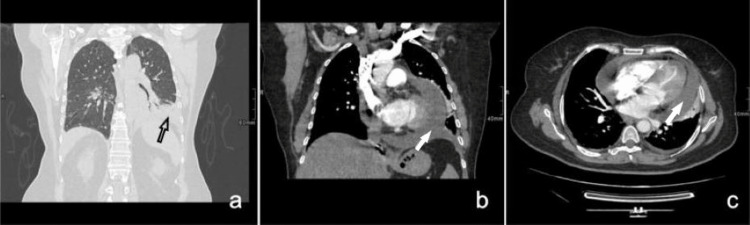
Chest x-ray and computed tomography show consolidation in the posterior and interior parts of the left lower lobe and the lingual of the left upper lobe (black arrow) (a). Large pericardial effusion is present (>3cm) (white arrows) (b,c).

The chest x-ray revealed infiltrates at the left lower lung lobe, pleural effusion of the left side, and an increased cardiothoracic index. A chest computed tomography (CT) with intravenous contrast was performed, and the presence of pericardial effusion was confirmed (> 3cm) (Figure 1). The patient was admitted to the cardiology department for further investigation and treatment.

The presence of mild PR segment depression was apparent at the electrocardiogram, and the trans-thoracic echocardiogram (TTE) revealed the presence of a large pericardial effusion causing tamponade and distention of the inferior vena cava with no respiratory variation. Due to an acute deterioration presenting with dyspnea and hypoxia [SpO_2_:91% (FiO_2_ 21%), pO_2_=53 mmHg, pH 7.5, lactate 0.8 mU/L], the patient was transferred to the intensive care unit where urgent pericardiocentesis was proposed, but the patient refused.

Empiric antibiotic therapy (vancomycin 1gr bid, ceftriaxone 2gr qd, levofloxacin 500mg qd, and metronidazole 500mg tid) was initiated in combination with methylprednisolone (20mg bid), colchicine 0.5mg bid and ibuprofen (600mg tid). The symptoms (fever, dyspnea), blood test values (inflammatory markers), and pericardial effusion were improved. The characteristic pericardial friction rub became prominent on the fifth day of admission.

Laboratory findings at admission are depicted in Table 1. Blood cultures (three samples taken at different time points) and urine cultures were negative.

**Table 1:** Abnormal patient’s laboratory findings at admission. ESR: Erythrocyte Sedimentation Rate, CRP: C-Reactive Protein, BNP: Brain Natriuretic Peptide, WBCs: White Blood Cells, LDH: Lactate Dehydrogenase

Laboratory findings	Patient’s results	Normal value
ESR	80 mm/h	< 20 mm/h
CRP	302 mg/L	< 5mg/L
Ferritin	706 μg/L	< 250 μg/L
BNP	271 pg/mL	< 100pg/mL
WBCs	12.300 /μL	< 10.000 /μL
Hemoglobin	7.5 gr/dL	≥ 12.0 gr/dL
Platelets	601.000 /μL	< 400.000 /μL
Serum protein	6.3 g/dL	6.5-8.3 g/dL
Albumin	2.2 g/dL	3.4-5.4 g/dL
LDH	280 IU/L	< 225 IU/L

**Figure 2:**
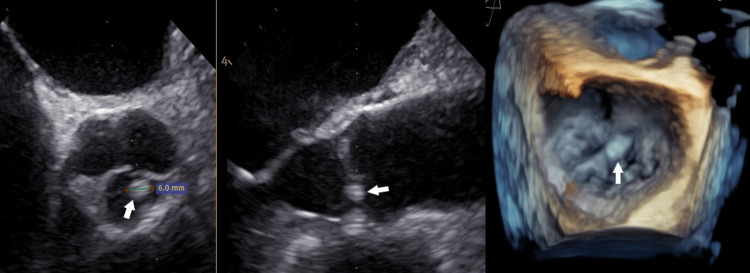
TEE revealed an echogenic finding of the right coronary cusp (white arrows).

Renal and hepatic functions were normal. Serology tests for coxsackie B1-B6, Cytomegalovirus, Herpes Simplex, Rubella, Varicella-Zoster, Epstein-Barr, Human Immunodeficiency Virus, Hepatitis-B, and Hepatitis-C were negative. Serum electrophoresis and serum immunofixation test were normal. A thoracentesis was performed for diagnostic purposes, where the pleural effusion was found to be transudate (protein 2.3g/dL, LDH 94IU/L, and glucose 153mg/dL) with negative cytology and culture. Thus, it was attributed to cardiac failure due to tamponade. The patient was found to have an increased titer of Anti-C. burnetii antibodies (IgG phases I and II and IgA phase I). Tests were re-conducted at the National Center of Infection Control with high titers of phase I IgG antibodies (>1:512), a result indicative of chronic C. burnetii infection. Although there was no previous animal contact in the last six months, the diagnosis of chronic Q fever was established, and the antimicrobial therapy was switched to doxycyclin 100mg bid. The patient became afebrile on the fifth day of treatment, whereas the laboratory findings were normal one week after treatment alteration. The patient was discharged one week later with monthly follow-ups where all laboratory investigations and chest x-rays were consistently normal. Due to the diagnosis of Q fever and a suspicion of an abnormal aortic valve finding in TTE, a trans-esophageal one (TEE) was performed, which revealed a significant reduction of the pericardial effusion, the presence of a minor mitral valve regurgitation but most importantly an echogenic region (maximum diameter of 6mm) of the right coronary cusp without aortic valve dysfunction, a finding compatible with possible endocarditis (Figure 2). The treatment was decided to be extended for 12 to 18 months to prevent disease recurrence.

## Discussion

Q fever was first described in 1937 when the causative pathogen was unknown. Since then, epidemic waves have occasionally been described, the worst of which was in the Netherlands in 2007 when 4000 patients became ill, and 20% of them were hospitalized due to airborne transmission of the disease from adjacent animal farms up to 5 km from the cities [4,5]. Originally classified as a member of the Rickettsia family, C. burnetti has nowadays considered a pathogen similar to Francisella and Legionella. It has a worldwide distribution and a wide variety of hosts ranging from mammals to arthropods. Both an acute and a chronic form of infection have been characterized. In the chronic form of the disease, the pathogen multiplies in the host’s macrophages and cause persistent bacteremia resulting in high titers of serum antibodies [6].

Diagnosis of Q fever is challenging in acute and chronic forms of infection. Laboratory findings in the acute form are non-specific, blood cultures are usually sterile, and diagnosis is via serology. Patients at high risk for Q fever (individuals with valvular or vascular diseases, immunodeficient patients, pregnant women, patients with a previous history of Q fever, individuals with a history of contact with farm animals and fever, patients with persistent and unexplained febrile episodes) should undergo repeated C. burnetii serologic testing [7]. In the chronic form, laboratory findings are more profound and can include: increased ESR, anemia, thrombocytopenia, elevated aminotransferases, and hematuria. Diagnosis is confirmed via serology [8], and the antigenic variation of the pathogen is used to differentiate acute from chronic form: the presence of IgG antibodies to phase II antigen indicates acute infection, while the presence of IgG antibodies to phase I antigen indicates chronic disease. The presence of IgM antibodies has been used with limited diagnostic value [9]. Due to the wide variety of symptoms and clinical presentations, as well as the delay between exposure to the pathogen and the onset of symptoms, the incidence of Q fever is probably underestimated. Q fever remains a poorly understood disease with various clinical manifestations ranging from subclinical to severe forms with fatal outcomes. Endocarditis is the most known and severe manifestation of chronic Q fever and can be fatal in 25-60% of cases [10].

Pericardial disease is often idiopathic, with a specific etiology found in less than 20% of patients [11,12]. C. burnetti is rarely considered a cause of pericardial disease, as very few Q fever cases presented with pericarditis have been reported [13-16], even the constrictive form [17]. Of these patients, a small percentage showed signs of cardiac tamponade and, indeed, within such a short period, as happened with our patient. In a large series, it has been suggested that C. burnetii may be the causative agent for 4.2% of total pericardial effusions and accounts for 6% of those previously characterized as idiopathic [18].

## Conclusions

Pericarditis is a rare clinical manifestation of Q fever. Despite the severity of complications, Q fever remains a treatable infection. However, a high degree of suspicion is usually required to reach a diagnosis as symptoms are non-specific, and a history of animal contact is not always evident. It is recommended to systematically test for Q fever in pericarditis cases of unknown etiology and unsatisfactory evolution, especially in areas where Q fever is endemic.
